# Effect of CFTR modulators on glucose homeostasis in children and young adults with cystic fibrosis-related diabetes: a systematic review

**DOI:** 10.3389/fendo.2025.1623654

**Published:** 2025-08-06

**Authors:** Paola Giordano, Giuseppina Leonetti, Vanja Granberg, Rosa Maria Pia Casolino, Giuseppe Lassandro, Maurizio Delvecchio, Giovanna Linguiti

**Affiliations:** ^1^ Department of Interdisciplinary Medicine, Pediatric Unit “B. Trambusti”, Cystic Fibrosis Regional Reference Center, University of Bari “Aldo Moro”, Bari, Italy; ^2^ Department of Biotechnological and Applied Clinical Sciences, University of L’Aquila, L’Aquila, Italy

**Keywords:** cystic fibrosis, CFTR modulators, CFRD, glucose metabolism, lumacaftor/ivacaftor, elexacaftor-ivacaftor-tezacaftor

## Abstract

**Introduction:**

Cystic fibrosis (CF) is an autosomal recessive disorder caused by mutations in the CFTR gene, leading to impaired chloride transport, thickened mucus, and multiorgan dysfunction. Among its complications, cystic fibrosis-related diabetes (CFRD) is a major concern, characterized by progressive b-cell dysfunction and insulin deficiency. The advent of CFTR modulators, including ivacaftor, lumacaftor/ivacaftor, and elexacaftor/tezacaftor/ivacaftor (ETI), has revolutionized CF management by improving pulmonary function, nutritional status, and overall survival. However, their effects on glucose metabolism remain under investigation.

**Methods:**

This systematic review (systematic review registration: PROSPERO 2025 CRD420251021499) analyzes recent evidence on the impact of CFTR modulators on CFRD in children and young adults. Results: Ivacaftor demonstrates potential benefits in glucose regulation, enhancing insulin secretion and glucagon control, particularly in patients with gating mutations. Conversely, lumacaftor/ivacaftor exhibits inconsistent effects, with some studies indicating glucose tolerance improvements while others report insulin sensitivity decline.

**Discussion:**

ETI therapy shows modest but generally positive effects on glycemic control, with reductions in HbA1c and fasting glucose, though without significant changes in insulin secretion. While CFTR modulators improve systemic health, their role in directly preventing or reversing CFRD remains unclear. Further longitudinal studies are needed to optimize therapeutic strategies and elucidate the long-term metabolic effects of CFTR modulation in CF patients.

**Systematic review registration:**

https://www.crd.york.ac.uk/PROSPERO/, identifier CRD420251021499.

## 1 Introduction

Cystic fibrosis (CF) is the most prevalent life-limiting autosomal recessive disorder among Caucasian populations, with an incidence of approximately 1 in 2,500–3,500 live births in Europe and North America (1). The disease results from mutations in the CFTR (Cystic Fibrosis Transmembrane Conductance Regulator) gene, located on chromosome 7q31.2, which encodes a chloride channel crucial for ion and fluid homeostasis across epithelial surfaces (2, 3). Dysfunctional CFTR activity disrupts ion transport, leading to the production of thick, dehydrated mucus that obstructs multiple organ systems, primarily affecting the respiratory, gastrointestinal, and reproductive tracts. CF manifests as a multisystemic disorder with highly variable phenotypic expression (4). The predominant clinical features involve the respiratory, gastrointestinal, endocrine, and reproductive systems. Respiratory complications include persistent cough, bronchiectasis, recurrent pulmonary infections (particularly with *Pseudomonas aeruginosa*) progressive decline in lung function, and eventual respiratory failure (5). Gastrointestinal involvement is characterized by pancreatic exocrine insufficiency, malabsorption, meconium ileus, distal intestinal obstruction syndrome (DIOS) (6), and hepatobiliary disease. Moreover, male patients frequently present with infertility due to congenital bilateral absence of the vas deferens (7). The pancreas is one of the earliest organs affected, with mucus accumulation leading to exocrine pancreatic insufficiency (EPI) due to progressive ductal obstruction, chronic inflammation, and acinar cell destruction. This results in fat malabsorption and deficiencies in fat-soluble vitamins (8), necessitating enzyme replacement therapy and dietary supplementation. In addition to exocrine dysfunction, pancreatic endocrine impairment progresses gradually, culminating in defective insulin secretion. Endocrine dysfunction commonly manifests as cystic fibrosis-related diabetes (CFRD) (7–9). CFRD exhibits characteristics of both type 1 diabetes (β-cell dysfunction) and type 2 diabetes (insulin resistance, particularly during pulmonary exacerbations) (9, 10) (**Table 1**). However, CFRD is marked by a progressive decline in insulin secretion with an often-subtle onset, underscoring the importance of early glucose monitoring.

**Table 1:** Main characteristics of the three types of diabetes.

FEATURE	CFRD	Type 1 Diabetes	Type 2 Diabetes
PRIMARY DEFECT	Insulin deficiency due to pancreatic fibrosis	Autoimmune β-cell destruction	Insulin resistance + β-cell dysfunction
AUTOANTIBODIES	Absent	Present	Absent
INSULIN RESISTANCE	Mild to moderate	Absent	Severe
KETOACIDOSIS RISK	Rare	High	Low
MANAGEMENT	Insulin therapy, nutritional support	Insulin therapy	Lifestyle, oral agents, insulin in advanced stages

CFRD primarily arises from insulin deficiency due to progressive destruction of pancreatic islets within a fibrotic and inflamed pancreas (11, 12). Over time, the inflammatory environment, compounded by fibrosis and ischemia, leads to islet damage, reducing β-cell mass and impairing insulin production (13). Even in preclinical stages, patients with cystic fibrosis frequently exhibit delayed and diminished insulin secretion, particularly in response to oral glucose intake. Although insulin deficiency is the principal mechanism underlying CFRD (14), insulin resistance also contributes (15), particularly during pulmonary exacerbations. Chronic inflammation in cystic fibrosis leads to elevated cytokine levels, such as TNF-α and IL-6, which interfere with insulin signaling (16). Additionally, frequent infections precipitate an increase in cortisol and catecholamines, further antagonizing insulin action. Nutritional status also modulates insulin sensitivity, as malnutrition reduces muscle mass (17) (a primary site for insulin-mediated glucose uptake) while corticosteroid use during exacerbations induces transient hyperglycemia.

Glucagon dysregulation and impaired incretin responses further contribute to CFRD pathophysiology. Some individuals exhibit abnormal glucagon secretion, exacerbating postprandial hyperglycemia. Additionally, deficiencies in incretin hormones, such as GLP-1 and GIP, further compromise insulin secretion and glucose homeostasis (18).

The prevalence of CFRD increases with age, affecting over 50% of patients older than 30 years (19, 20). Its clinical impact is profound, being associated with accelerated pulmonary decline, heightened infection susceptibility, nutritional deterioration, and increased mortality.A customized and individualized insulin therapy remains the recommended treatment for children and adolescents with CFRD (21), whereas there is only limited evidence for use of oral hypoglycemic agents (22). Beyond glycemic regulation, insulin therapy plays a pivotal role in improving nutritional status and pulmonary function. Nutritional support is crucial, with dietary recommendations emphasizing a high-calorie, high-fat intake (22), supplemented with pancreatic enzyme replacement therapy (PERT) (23) and fat-soluble vitamins to optimize nutrient absorption. Regular physical activity further enhances metabolic stability by improving insulin sensitivity, increasing muscle mass (24), and promoting respiratory function.

The advent of CFTR modulators represents a landmark advancement in cystic fibrosis therapy. They are administered orally, typically twice daily with fat-containing meals to optimize absorption. Treatment initiation necessitates genotypic confirmation of CFTR mutations and vigilant monitoring for potential adverse effects, including hepatotoxicity and drug interactions. Given their profound impact on disease progression, CFTR modulators are now considered standard therapy for eligible patients. Unlike conventional treatments aimed at symptomatic relief, these agents directly target the underlying molecular defect caused by CFTR gene mutations.

CFTR modulators are categorized into 1. potentiators and 2. correctors. The most advanced combination, elexacaftor/tezacaftor/ivacaftor (ETI, marketed as Trikafta/Kaftrio), has demonstrated substantial benefits, including improved lung function, reduced exacerbation frequency, and enhanced nutritional status in patients harboring at least one F508del allele (25–28). Their introduction has significantly enhanced pulmonary function, nutritional status, and life expectancy in eligible patients (29). Notably, CFTR modulators have been associated with improvements in pancreatic function, leading to enhanced fat and vitamin absorption and a reduced reliance on dietary supplementation (30). However, these benefits are not universally experienced.

This literature review synthesizes recent findings on the efficacy of CFTR modulators on metabolic control, nutritional outcomes and the treatment of Children and Young Adults with CFRD.

## 2 Materials and methods

### 2.1 Search strategy

We searched electronic databases (Pubmed and Web of Science) for studies published between 1^st^ January 2010 (the year the first study was published) and 31th December 2024. Search terms, or “MESH” (MEdical Subject Headings) for this systematic review included various combinations: “hyperglycemia” or “diabetes” or “glucose disorder” AND “cystic fibrosis” AND “elexacaftor” OR “Ivacaftor” OR “Tezacaftor”. To avoid missing any relevant studies, we also screened the reference list of eligible studies. We formulated 5 questions related to the efficacy of the CFTR modulators and for each one we established the outcomes listed below. The protocol was registered with the International Prospective Register of Systematic Reviews database (PROSPERO https://www.crd.york.ac.uk/PROSPERO, number CRD420251021499).

### 2.2 Criteria for study selection

We conducted a systematic search of the literature based on the PICOS model (Population, Intervention, Comparison, Outcomes, Study design) (**Table 2**).

**Table 2:** PICOS model.

Population	Children (birth – 18 years) and young adults (19–24 years) with cystic fibrosis-related diabetes (CFRD)
Intervention	CFTR modulators (e.g., ivacaftor, lumacaftor/ivacaftor, elexacaftor/tezacaftor/ivacaftor)
Comparison	Standard CFRD management without CFTR modulators or placebo
Outcomes	Changes in glucose metabolism, insulin secretion, glycemic control and progression of CFRD
Study design	Randomized clinical trials (RCTs), observational studies (cohort, case-control), and experimental research

Inclusion criteria were as follows: i) Study population: Children (birth to 18 years) and young adults (19 to 24 years) with hyperglycemia or cystic fibrosis-related diabetes (CFRD) who received CFTR modulator therapy, with available follow-up data; ii) Study design: Observational studies (cohort, case-control), exploratory studies, and experimental research; iii) Review articles were excluded, but their reference lists were screened for potentially eligible studies; iv) Only full-text papers were included; abstracts only were excluded; v) Data on therapy efficacy: Information on the age at the start of therapy, duration of treatment, and outcomes; vi) Publication date: Studies published in the last 15 years (2010–2024).

Exclusion criteria included: i) Studies with data available without baseline status (cross-sectional studies); ii) Studies with only baseline data, without follow-up; iii) Animal studies; iv) Full-text not available; v) studies not yet published; vi) Studies not reporting the selected outcomes; vii) Studies on CFRD without data on CFTR modulator therapy; viii) Studies in which data concerning the population of interest cannot be extracted; ix) Studies published in languages other than English were not excluded *a priori*.

### 2.3 Data extraction and management

Two independent investigators (VG, GL) screened for inclusion the title and abstract of all the studies identified using the search strategy. Any discrepancies between them were resolved by consensus. After abstract selection, the same two investigators conducted the full paper analysis.

The following characteristics were evaluated for each study in the full paper: i) reference details: authorship(s); published or unpublished; year of publication; period in which the study was conducted; other relevant cited papers; ii) study characteristics: study design, topic, treatment period, follow up duration, region; iii) population characteristics: number of participants, age and demographic data; comparator characteristics; iv) methodology: measures to assess the outcomes; v) main results: outcome measures.

### 2.4 Characteristics of included studies

**Figure 1:**
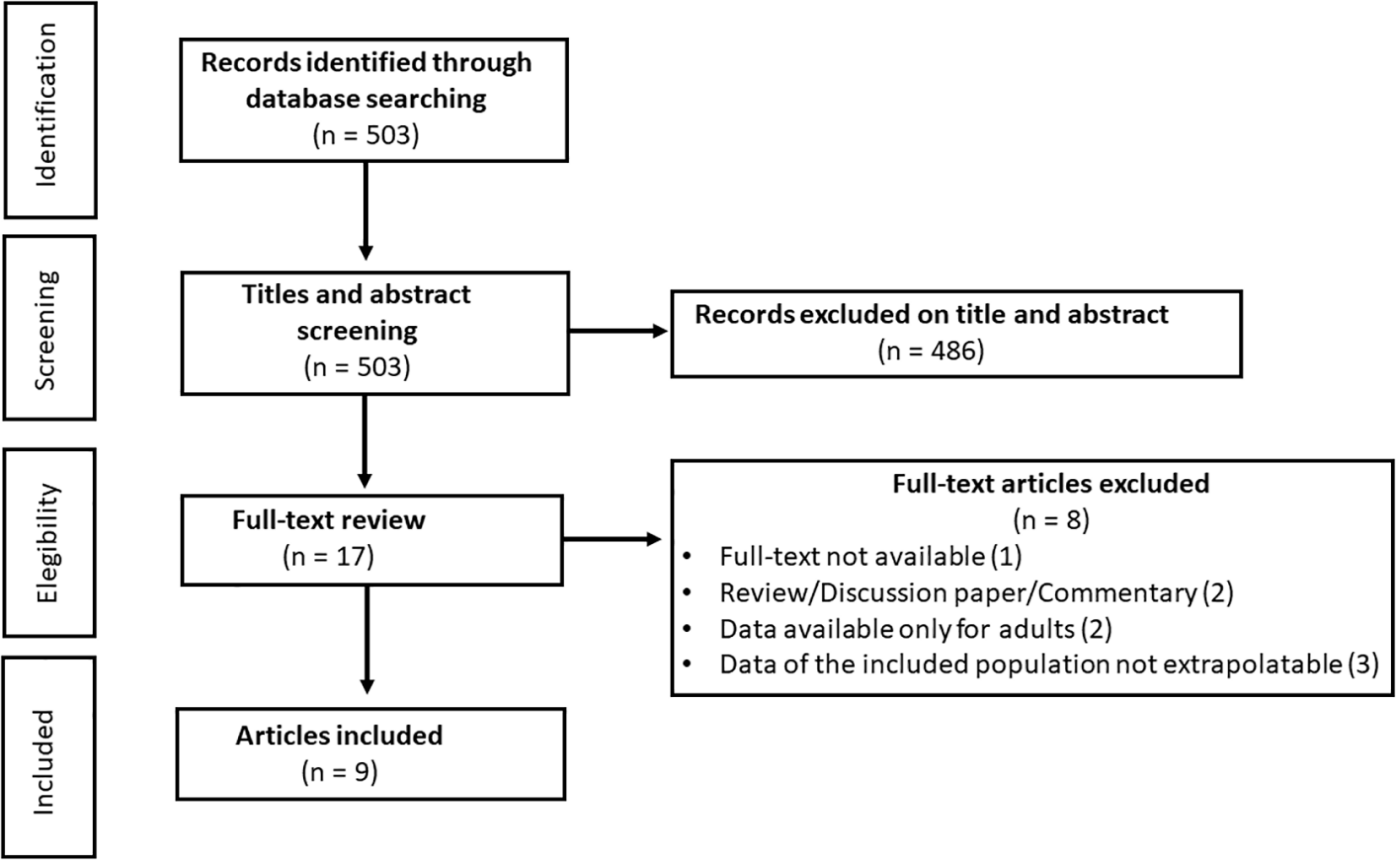
Flow chart of study inclusion in systematic review.

After removal of duplicate, the titles and abstract of 503 unique references were evaluated for eligibility. A total of 486 were excluded, and 17 full-text articles were extracted and screened for eligibility. Of these, 9 (31–39) original studies met eligibility criteria (**Figure 1**). Of these studies, 4 were conducted in the United States of America, 1 in Italy, 2 in Israel, 1 in France and 1 in Germany (**Table 3**).

**Table 3:** Characteristic table.

Author and year	Country	Age range	Sample size	Study design	Drug molecule	Measured parameters	Outcomes	Conclusion
Bassi et al., 2024 (31)	Italy	12-56	28	Retrospective observational	Elaxacaftor-Tezacaftor-Ivacaftor	HbA1c, TDD, FEV1, FVC, BMI, number of hospitalizations and pulmonary exacerbations, CFQ-R Data collected at four time points	Improved glycemic control: HbA1c levels decreased at all time points. Reduced insulin requirement: TDD decreased at 3 and 6 months, but not significantly at12 months. Improved pulmonary function: FEV1% and FVC significantly increased at 3, 6, and 12 months. Fewer hospitalizations and pulmonary exacerbations. Increased BMI, indicating improved nutritional status. Better quality of life. No patients stopped insulin therapy after starting ETI. in heterozygous F508del/other patients, insulin requirements did not decrease, but HbA1c improved.	ETI therapy led to better glycemic control in insulin-dependent CFRD patients, shown by reduced HbA1c. Insulin requirements decreased in the first 6 months, but no further reduction was observed at 12 months. Significant improvements in pulmonary function, nutritional status, and quality of life were confirmed. More studies with longer follow-ups and larger samples are needed to fully understand ETI’s role il glucose regulation in CFRD patients.
Chan et al., 2024 (32)	USA	≥ 12	79	Prospective observational multicenter	Elaxacaftor-Tezacaftor-Ivacaftor	Fasting and postprandial glucose levels, HbA1c, insulin secretion rates, insulin sensitivity, FEV1, BMI	After 24–30 months of ETI therapy, fasting glucose decreased slightly (from 94 mg/dL to 90 mg/dL, p = 0.02) in patients not on insulin therapy. HbA1c significantly decreased (from 5.8% to 5.5%, p < 0.001), suggesting improved glycemic control. Insulin sensitivity decreased over time (p = 0.03), but insulin secretion remained stable. Glucose tolerance varied among patients: some showed improvements, others worsened, and some remained stable.	After two yeras of ETI therapy, there were moidest improvements in fasting glucose and HbA1c, but glucose tolerance did not change consistently. Insulin secretion adjusted to the decrease in insulin sensitivity, preventing overall deterioration on β-cell function. ETI may help stabilize or slow down the decline in β-cell function, but it is unclear wheter it can prevent or reverse CFRD. Further long-term studies, especially in children, are needed to assess the early impact of ETI on glucose metabolism.
Li et al., 2019 (33)	USA	≥ 12	9	Observational	Lumacaftor-Ivacaftor	CGM, HbA1c, glucose tolerance, BMI, FVC, FEV1	After treatment, increase in HbA1c and fasting glucose were observed (p= 0.02). No significant changes were detected in CGM parameters or in glucose level at 1 and 2 hours post-OGTT. Sex differences: males exhibited reduced glycemic variability post-treatment, while females showed no significant changes.	Glycemic abnormalities persisted in CF patients treated with Lumacaftor-Ivacaftor. Sex-related differences may influence glycemic response to treatment. Further research, particularly in younger patients, is needed to evaluate the long-term impact of Lumacaftor-Ivacaftor on glucose metabolism and the progression of CFRD.
Misgault et al., 2020 (34)	France	≥ 12	40	Prospective multicenter observational	Lumacaftor-Ivacaftor	Glucose tolerance, fasting glucose, insulin, C-peptide, BMI, albumin levels, FEV1, FVC	After 1 year of treatment glucose tolerance improved in 57.5% of patients (50% achieved NGT – compared to 0% at baseline -, 40% had IGT – vs 78% at baseline – only 10% remained CFRD – vs 22% at baseline-). Significant reduction in 2-hours OGTT glucose levels: from 171 mg/dl (baseline) to 139 mg/dl (post-treatment) (p < 0.001). No significant changes in insulin or C-peptide levels. BMI and albumin levels significantly increased, suggesting improved nutritional status. FEV1 and FVC significantly improved, indicating better pulmonary function.	Lumacaftor/Ivacaftor imrpoved glucose tolerance in a significant proportion of CF patients. The improvement in glucose metabolism was not associated with increased insulin secretion, suggesting an indirect effect rather than a direct impact on pancreatic β-cell function. The beneficial metabolic effects may be related to improvements in overall nutritional and respiratory status rather than a direct action on CFTR function in β-cells. Larger and randomized trials are needed to further investigate the role of CTFR modulators in glucose metabolism and CFRD prevention.
Thomassen et al., 2018 (35)	Germany	≥ 12	5	Observational	Lumacaftor-Ivacaftor	Glucose metabolism, insulin secretion, BMI, sweat chloride concentration, FEV1	After 6–8 weeks of treatment, three patients showed improved 2-hour glucose levels, while two experienced worsening. OGTT glucose curve analysis revealed an increase in the AUC in three out of five patients. In response tto IVGTT, insulin secretion improved in two patients but worsened in three. All patients experienced weight gain and an increase in BMI. Sweat chloride concentration decreased in four out of five patients, indicating improved CFTR function.	The study did not demonstrate a consistent impact of Lumacaftor/Ivacaftor therapy on glucose tolerance or insulin secretion in CF patients homozygous for Phe508del. Treatment responses were highly variable. The authors emphasize the need for larger studies with extended follow-up to better assess the drug’s effects on endocrine pncreatic function and its potential role in preventing CFRD.
Tsabari et al., 2016 (36)	Israel	22-24	2	Case-report	Ivacaftor	Glucose tolerance, AUC for glucose and insulin	Case 1 (indeterminate glycemia) improved early insulin secretion, reducing glucose AUC by 12.35%. Case 2 (CFRD) increased insulin AUC by 77%, with glucose AUC reduced by 16%. Diabetes status improved from CFRD to indeterminate glycemia. Both patients showed enhanced glucose metabolism, with increased early-phase insulin secretion and better glycemic response to OGTT.	Ivacaftor improved early-phase insulin secretion in CF patients carrying the S549R mutation, leading to the resolution of CFRD in one case. The improvement in insulin secretion may be mediated by CFTR reactivation in pancreatic β-cell. Further studies with larger cohorts are needed to confirm these findings and evaluate Ivacaftor’s efficacy in other CFTR mutations.
Dagan et al., 2017 (37)	Israel	12-40	8	Retrospective	Ivacaftor	FEV1, FVC, FEF25-75%, sweat chloride concentration, BMI, glucose metabolism, HbA1c, pseudomonas aeruginosa colonization, use of intravenous antibiotics.	Significant improvement in polmonary function: FEV1 increased from 74% to 88% (p < 0.001); FVC increased from 89% to 101% (p = 0.019); FEF25-75% increased from 59% to 76% (p = 0.019). Sweat chloride concentration decreased from 116 ± 8 mmol/L to 51 ± 17 mmol/L (p < 0.001); BMI increased from 20 ± 3 to 22 ± 4 (p = 0.003), improvement in glucose metabolism in 5 patients: 2 patients with CFRD shifted to IGT; 3 patients with IGT normalized to NGT; no significant changes in pseudomonas aeruginosa colonization. Reduction in the use of intravenous antibiotics.	Ivacaftor led to significant clinical improvements in CF patients carrying the p.Ser549Arg mutation, including: Better pulmonary function, restoration of CFTR function, improved BMI and glucose metabolism. The drug did not eliminate chronic infections but reduced the need for intravenous antibiotics. This study supports the efficacy of Ivacaftor for this rare mutation, reinforcing its clinical use.
Bellin et al., 2013 (38)	USA	6-52	5	Observational Open-label pilot	Ivacaftor	Insulin secretion, blood glucose levels, changes in glucose tolerance category, BMI	Improved insulin secretion after one month of ivacaftor therapy; Specific improvements in patients with insulin dysfunction: the patient with IGT transitioned to NGT. Partial restoration of insulin secretion in patients who had no detectable insulin response at baseline. No significant changes in blood glucose levels in patients with CFRD. Average BMI increase of 0.5 kg/m^2^ among partecipants.	Ivacaftor improves insulin secretion in CF patients, suggesting a direct role of CFTR in β-cell function. The drug may slow down or prevent diabetes progression, particularly if initiated early. Larger and long-term studies are needed to confirm these effects and evaluate whether early CFTR correction could delay or prevent diabetes in CF patients.
Kelly et al., 2019 (39)	Pennsylvania	≥ 6	12	Longitudinal observational	Ivacaftor	Glucose tolerance, insulin secretion, incretin secretion, glucagone secretion, FEV1	Improved insulin secretion: after 4 months of Ivacaftor, insulin release in response to arginine was significantly enhanced under both fasting and glucose-stimulated conditions. Increased β-cell response. The sidpodition index, which measures insulin secretion relative to insulin sensitivity, improved significantly (p=0.04). Better glucagon control: glucagon suppression in response to insulin was more effective post-treatment. No significant improved in glucose tolerance: Fasting and post-prandial glucose levels remained unchanged. No change in incretin secretion (GLP-1 and GIP)	Four months of Ivacaftor therapy improved β-cell function, enhancing insulin secretion and glucagon control, but did not significantly alter glucose tolerance or incretin secretion. These findings suggest that Ivacaftor may help prevent CFRD, but long-term studies are needed to confirm these effects.

## 3 Results

### 3.1 Ivacafactor

Ivacaftor appears to exert a direct effect on insulin secretion. More than 10 years ago, Bellin et al. (2013) (38) reported the first data about this drug showing that it may improve insulin secretion in a one-month study. Notably, one patient with impaired glucose tolerance achieved normoglycemia, while the others exhibited partial restoration of insulin secretion. Unfortunately, in patients with established CFRD, no significant changes in blood glucose levels were observed. This study suggested for the first time that Ivacaftor may be effective in preventing diabetes progression if initiated early, but not in reversing CFRD once it has developed.

Further, Tsabari et al. (2016) (36) investigated its effects in two CF patients with glucose abnormalities and found that both demonstrated improved insulin secretion, with one patient transitioning from CFRD to an intermediate glycemic status. These findings confirmed that Ivacaftor could contribute to β-cell function restoration, suggesting that even in patients with CFRF blood glucose can improce.

Dagan et al. (2017) (37) demonstrated that some patients carrying the p.Ser549Arg mutation treated with Ivacaftor experienced enhanced glucose metabolism, with transitions from CFRD to impaired glucose tolerance and from impaired glucose tolerance to normoglycemia.

Finally, Kelly et al. (2019) (39) confirmed that Ivacaftor enhances β-cell function and glucagon regulation without significantly altering glucose tolerance or incretin secretion. These findings indicate that Ivacaftor may support pancreatic function but does not fully correct the metabolic abnormalities associated with CF.

### 3.2 Lumacaftor/ivacaftor

The effects of Lumacaftor/Ivacaftor on glucose metabolism have been explored in multiple studies, with inconsistent findings. On one hand, Li et al. (2019) (33) observed an increase in HbA1c and fasting glucose levels following treatment, with no significant changes in continuous glucose monitoring (CGM) parameters. Moreover, sex differences were identified, with male patients exhibiting reduced glycemic variability, while female patients showed no significant changes. This study suggests that Lumacaftor/Ivacaftor does not effectively correct glycemic abnormalities in CF patients and that individual factors may influence metabolic responses to treatment.

In contrast, Misgault et al. (2020) (34) reported that 57.5% of CF patients treated with Lumacaftor/Ivacaftor experienced an improvement in glucose tolerance after one year of therapy. Specifically, a greater proportion of patients achieved normal glucose tolerance, while fewer persisted with CFRD. However, this improvement was not associated with increased insulin secretion, suggesting that the observed metabolic benefits were likely secondary to enhancements in nutritional and respiratory status rather than a direct effect on pancreatic β-cell function.

Thomassen et al. (2018) (35) did not observe significant improvements in glucose regulation following Lumacaftor/Ivacaftor treatment. However, some patients experienced weight gain and an increase in BMI, suggesting a potential nutritional benefit.

### 3.3 Elexacaftor-tezacaftor-ivacaftor

Conversely, Bassi et al. (2024) (31) reported that treatment with ETI led to improved glycemic control, as evidenced by a reduction in HbA1c levels across all study time points. Additionally, a decrease in insulin requirements was observed during the first six months of therapy, although no further reduction was noted at 12 months. Patients receiving ETI also exhibited significant improvements in pulmonary function, a reduction in hospitalizations and pulmonary exacerbations, increased BMI, and an overall enhancement in quality of life. However, no patient was able to discontinue insulin therapy, suggesting that while ETI improves glycemic control, it is not sufficient to normalize glucose metabolism in CFRD patients.

Chan et al. (2024) (32) investigated the effects of ETI over a longer period (24–30 months). Their findings revealed a modest decrease in fasting glucose levels and a significant reduction in HbA1c, indicating improved glycemic control. However, insulin sensitivity declined over time, and glucose tolerance exhibited variable responses among patients—some showed improvement, others worsened, and some remained stable. These data suggest that ETI may help stabilize or slow the decline of β-cell function, but whether it can prevent or reverse CFRD remains uncertain.

## 4 Discussion

CFTR modulators have revolutionized the treatment landscape for cystic fibrosis CF by targeting specific genetic mutations and restoring defective CFTR protein function. While these therapies have significantly improved pulmonary function and nutritional status, their effects on glucose metabolism and CFRD remain an area of active investigation.

Ivacaftor has demonstrated significant metabolic benefits in patients with gating mutations such as G551D and S549N (**Table 4**). Clinical studies indicate improved glucose metabolism, with increased insulin secretion and reduced postprandial glucose levels (36–39). These effects likely stem from enhanced pancreatic duct function, reducing exocrine pancreatic stress and creating a more favorable endocrine environment. Ivacaftor has also been associated with improved glucose tolerance, particularly in patients treated early in disease progression (36–39). The direct role of CFTR in pancreatic β-cell function has been suggested, given that Ivacaftor increases early-phase insulin secretion and enhances glucagon suppression (38, 39), which may slow CFRD progression.

**Table 4:** Relationship between CFTR mutations and modulator efficacy.

Mutation class	Example mutations	Effective modulator	Expected response
I - No synthesis	G542X, W1282X	None (current modulators ineffective)	No response
II - Trafficking defect	F508del	Lumacaftor/Ivacaftor, ETI	Good response with ETI
III - Gating defect	G551D, S549N	Ivacaftor	Excellent response
IV - Conductance defect	R117H	Ivacaftor	Moderate improvement
V - Reduced expression	3849 + 10kb C→T	Ivacaftor (variable)	Mild to moderate benefit

In addition to metabolic benefits, Ivacaftor significantly enhances pulmonary function, with FEV1 improvements exceeding those observed with other modulators (37). FVC and BMI also increase, likely due to improved nutrient absorption and overall better health status. However, its efficacy is largely limited to patients with Class III (gating) and some Class IV (conductance) mutations, with minimal to no effect in Class I and II mutations, which involve defective CFTR synthesis or trafficking (40). Lumacaftor/Ivacaftor has produced mixed results regarding glucose metabolism. Some studies suggest improvements in glucose tolerance (34), while others indicate no significant changes or even worsening glycemic control (33, 41, 42). Insulin secretion remains largely unchanged, suggesting that any metabolic improvements are indirect, likely resulting from reduced systemic inflammation and improved overall health rather than direct β-cell restoration. Contrary to expectations, Piona et al. demonstrated that insulin sensitivity worsened in CF patients treated with lumacaftor/ivacaftor (43).

Pulmonary function benefits are moderate but less pronounced than those observed with Ivacaftor or ETI. FEV1 increases are modest, and FVC shows slight improvements. BMI also exhibits a mild to moderate increase, likely secondary to improved pulmonary status. However, the drug’s tolerability issues and its limited efficacy in heterozygous F508del patients have restricted its widespread adoption. ETI therapy has significantly expanded treatment options for CF patients carrying at least one F508del allele. Its effects on glucose metabolism appear modest but generally positive, with reductions in HbA1c and slight decreases in fasting glucose (31, 32). While insulin secretion shows mild increases in some patients, overall β-cell function remains largely unchanged. Notably, glucose tolerance does not consistently improve, and CFRD progression does not appear to be significantly altered. Beyond glucose metabolism, ETI produces the most substantial improvements in pulmonary function among all CFTR modulators, with FEV1 increases of 10–15% and a reduction in pulmonary exacerbations. FVC improves, particularly in patients with advanced respiratory impairment. BMI increases considerably, reflecting enhanced nutrient absorption and improved pulmonary status. According to Grancini et al. (44), initiation of ETI therapy was linked to better glycaemic control in insulin-treated CFRD patients. Similarly, Bassi et al. demonstrated an improvement in glycaemic control accompanied by a significant reduction in insulin requirements in a cohort of both pediatric and adult patients with CFRD (31). Given its efficacy in patients with at least one F508del allele, ETI represents the most versatile and broadly effective CFTR modulator currently available. The effectiveness of CFTR modulators is closely linked to the type of CFTR mutation present. Ivacaftor is highly effective in Class III (gating) mutations, where it enhances CFTR channel opening, while it has minimal impact on Class I and II mutations. Lumacaftor/Ivacaftor provides moderate benefits to homozygous F508del patients by improving CFTR protein trafficking, although its metabolic effects remain inconsistent. ETI, by contrast, offers substantial clinical benefits to a broader CF population, including those with at least one F508del allele (**Table 4**).

While CFTR modulators have revolutionized CF care, their metabolic effects, particularly in the context of CFRD, remain an area requiring further research. Ivacaftor demonstrates the most direct benefits on β-cell function, whereas Lumacaftor/Ivacaftor and ETI appear to exert indirect metabolic effects, likely mediated through systemic improvements in health. Future research should focus on addressing treatment gaps, particularly for Class I mutations, through emerging therapies such as gene editing and read-through compounds.

CFTR modulators have transformed CF treatment, but their impact on glucose metabolism and CFRD progression remains an area of active investigation. While Ivacaftor appears to have a direct positive impact on insulin secretion, Lumacaftor/Ivacaftor and ETI seem to exert more indirect metabolic effects, likely mediated by improvements in nutritional and respiratory status. The findings remain heterogeneous, highlighting the need for further research to fully elucidate the role of CFTR modulators in the prevention and management of CFRD.

A deeper understanding of the interplay between CFTR function and pancreatic endocrine regulation will be essential in developing targeted therapies that not only improve pulmonary health but also address metabolic dysfunction in CF patients.

The variability in metabolic responses highlights the complexity of CFRD pathophysiology and the need for personalized treatment approaches. Future research should focus on early intervention strategies, particularly in young patients, to assess if CFTR correction can delay β-cell deterioration. Next-generation CFTR modulators and emerging genetic therapies, such as gene editing and RNA-based approaches, may offer new avenues for more effective CFRD management.

## Data Availability

The original contributions presented in the study are included in the article/supplementary material. Further inquiries can be directed to the corresponding author/s.
